# Effects of Sorafenib on *C*-Terminally Truncated Androgen Receptor Variants in Human Prostate Cancer Cells

**DOI:** 10.3390/ijms130911530

**Published:** 2012-09-14

**Authors:** Friedemann Zengerling, Wolfgang Streicher, Andres J. Schrader, Mark Schrader, Bianca Nitzsche, Marcus V. Cronauer, Michael Höpfner

**Affiliations:** 1 Department of Urology, Ulm University, Ulm 89075, Germany; E-Mails: ajschrader@gmx.de (A.J.S.); mark.schrader@uniklinik-ulm.de (M.S.); marcus.cronauer@uni-ulm.de (M.V.C.); 2 Department of Physiology, Charité Universitätsmedizin, Campus Benjamin Franklin, Berlin 14195, Germany; E-Mails: bianca.nitzsche@charite.de (B.N.); michael.hoepfner@charite.de (M.H.); 3 Institute of General Zoology and Endocrinology, Ulm University, Ulm 89069, Germany; E-Mail: wolfgang.streicher@uni-ulm.de

**Keywords:** sorafenib, truncated androgen receptor variants, castration resistant prostate cancer

## Abstract

Recent evidence suggests that the development of castration resistant prostate cancer (CRPCa) is commonly associated with an aberrant, ligand-independent activation of the androgen receptor (AR). A putative mechanism allowing prostate cancer (PCa) cells to grow under low levels of androgens, is the expression of constitutively active, *C*-terminally truncated AR lacking the AR-ligand binding domain (LBD). Due to the absence of a LBD, these receptors, termed ARΔLBD, are unable to respond to any form of anti-hormonal therapies. In this study we demonstrate that the multikinase inhibitor sorafenib inhibits AR as well as ARΔLBD-signalling in CRPCa cells. This inhibition was paralleled by proteasomal degradation of the AR- and ARΔLBD-molecules. In line with these observations, maximal antiproliferative effects of sorafenib were achieved in AR and ARΔLBD-positive PCa cells. The present findings warrant further investigations on sorafenib as an option for the treatment of advanced AR-positive PCa.

## 1. Introduction

Prostate cancer (PCa) is the most common neoplasm and the third leading cause of cancer-related deaths in elderly men of the western world [1]. Localized PCa is treatable and potentially curable by radical prostatectomy or radiation therapy. As most PCa cells depend on androgens for growth and survival, current treatment for non-organ confined PCa is mainly based on androgen deprivation (AD) like surgical or chemical castration and/or systemic administration of anti-androgens. Although the majority of PCa initially responds well to AD, complete remissions are rare and most tumors recur in a more aggressive form that does no longer respond to endocrine therapies. For this stage of the disease, also designated as castration resistant PCa (CRPCa), treatment options are limited and palliative.

Continued androgen receptor (AR)-signalling remains the dominant growth pathway in prostate cancer progressing under low levels of circulating androgens [2]. Various mechanisms have been described to explain the aberrant activation of AR-mediated signalling in CRPCa cells: These include AR-gene amplification and/or overexpression of AR-protein (hypersensitive pathway), point mutations that broaden ligand-specificity of the AR (promiscuous pathway), AR-activation by peptide growth factors or cytokines (outlaw pathway) as well as intratumoral steroid synthesis (backdoor pathway) [3,4]. Synthesis of *C*-terminally truncated AR-variants has emerged as an important mechanism of CRPCa cells to grow and survive under subphysiological levels of circulating androgens [5–7]. As the ligand binding domain (LBD) of the AR is localized in the *C*-terminus, these AR-variants are referred to as ARΔLBD. Several molecular mechanisms enabling CRPCa cells to synthesize ARΔLBD have been identified: Most ARΔLBD found in cell lines and tissue specimens of metastatic PCa are products of alternative splicing (AR-V) [5,8,9]. However, nonsense mutations in the LBD or hinge region of the AR (AR^Q640X^) [6] as well as enzymatic cleavage of the AR protein [7,10] have also been described to generate ARΔLBD. In contrast to full length AR, which translocates into the nucleus upon androgenic stimuli, many ARΔLBD are able to enter the nucleus even in the absence of androgens [5,6]. Although ARΔLBD largely vary in their synthesis, a recently described core domain consisting of the AR *N*-terminal domain and the DNA-binding domain (NTD/DBD core) is sufficient for AR-variants to access the nucleus and to activate AR-target genes [11]. Due to the absence of a functional LBD, the constitutively active ARΔLBD are insensitive towards classical endocrine therapies, which either directly target the LBD (antiandrogene therapies) or indirectly target LBD-function by suppression of androgen synthesis. Moreover, aberrant reactivation of AR-signalling by ARΔLBD has recently been suggested to contribute to resistance to anti-hormonal treatments with CYP17 inhibitors, such as abiraterone [12]. Thus, there is an urgent need for new therapies targeting ligand-independent AR-signalling in CRPCa cells to expand the therapeutic options for the treatment of advanced PCa.

AR-function as well as AR-stability largely depend on post-translational modifications like phosphorylation on serine/threonine or tyrosine residues [13,14]. Recently, Oh *et al*. reported that the multikinase inhibitor sorafenib (Nexavar, BAY 43-9006) was able to decrease proliferation of PCa cells via inhibition of the canonical AR-signalling pathway. Inhibition of AR-signalling was paralleled by a downregulation of AR protein-levels [15]. As most AR phosphorylation sites are located at the *N*-terminus [13], a region shared by both, full length AR and ARΔLBD, we hypothesized that sorafenib might also affect ARΔLBD-function. Therefore, we investigated the effect of sorafenib on ARΔLBD-signalling using AR-negative PC-3 cells transiently transfected with the ARΔLBD-variant AR^Q640X^ as well as the AR/AR-V expressing PCa cell line 22Rv1 as experimental models.

## 2. Results and Discussion

### 2.1. Sorafenib Inhibits Canonical AR-Signalling in PCa Cells

**Figure 1:**
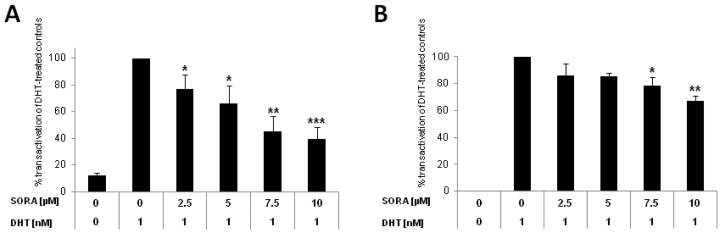
Sorafenib inhibits androgen receptor (AR)-signalling in prostate cancer (PCa) cells. PC-3 cells were cotransfected with an AR-expression construct together with an AR-dependent (**A**) Prostate specific antigen (PSA) or (**B**) ARE(2x)-reporter plasmid. pRL-tk-LUC was co-transfected as an internal control for transfection efficiency. Reportergene activity after sorafenib treatment (SORA) was measured using a Dual-Luciferase Reporter Assay as recently described [17]. Results are expressed in percent transactivation of dihydrotestosterone (DHT)-treated cells which were set at 100%; * *p* < 0.05; ** *p* < 0.01; *** *p* < 0.001.

Recently, sorafenib was shown to target AR-signalling in LNCaP and castration resistant LNCaP-sublines (LNCaP-abl, LNCaP-IL6+, LNCaP-Bic) [15]. As LNCaP cells express a promiscuous full length AR (919 amino acids, point mutation in the LBD, T877A) [16], we analyzed the effects of sorafenib on AR-signalling in PC-3 cells transiently transfected with a wild type AR. As seen in Figure 1, sorafenib diminished transactivation of AR-dependent reportergene constructs (ARE(2x), PSA) in PC-3 cells. Inhibition was already significant for the PSA-reporter at a concentration of 2.5 μM (downregulation *versus* DHT-treated controls: 23% ± 11%, *p* = 0.034), reaching its maximum at 10 μM, (downregulation *versus* DHT-treated controls: 60% ± 10%, *p* < 0.001; Figure 1A). Inhibition of ARE(2x)-promoter construct was relatively weak but statistically significant at concentrations >5μM (downregulation *versus* DHT-treated controls at 7.5 μM, 22% ± 6%, *p* = 0.033) reaching its maximum at 10 μM (downregulation 32% ± 4%, *p* = 0.002; Figure 1B). The reportergene assays are in agreement with previous findings, showing a sorafenib-induced downregulation of prostate specific antigen (PSA) in LNCaP and castration resistant LNCaP sublines [15].

### 2.2. Effect of Sorafenib on Constitutively Active, C-Terminally Truncated AR-Mutant Q640X

Posttranslational modifications like phosphorylation on serine, threonine or tyrosine residues are involved in a large variety of steroid receptor functions [13,14]. Based on recent findings by Oh *et al*. it is tempting to speculate that the multikinase-inhibitor sorafenib targets the AR phosphorylation via blockade of a yet undefined kinase [15]. As the majority of AR phosphorylation sites are located at the *N*-terminus of the receptor molecule, we hypothesized that sorafenib might also affect phosphorylation of the ARΔLBD-*N*-terminus.

**Figure 2:**
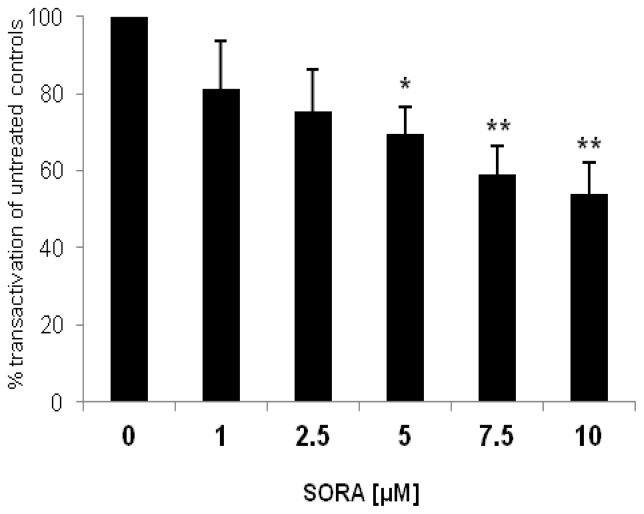
Sorafenib inhibits AR^Q640X^-signalling in PC-3 cells. AR negative PC-3 cells were cotransfected with an AR^Q640X^ construct (AR with point mutation in the hinge region, 640 amino acids) together with an ARE(2x)-reporter plasmid. pRL-tk-LUC was co-transfected as an internal control for transfection efficiency. Reportergene activity after sorafenib treatment (SORA) was measured using a Dual-Luciferase Reporter Assay as recently described [17]. Results are expressed in percent transactivation of untreated controls which were set at 100%; * *p* < 0.05; ** *p* < 0.01.

As an experimental model we transfected PC-3 cells with the AR-mutant AR^Q640X^, the product of a nonsense mutation leading to a stop codon in the hinge region adjacent to the LBD of the AR [6]. Transactivational activity of AR^Q640X^ was shown to be very strong on artificial androgen-responsive promoters (ARE(2x)) but was very weak on the PSA promoter [17,18]. In contrast to the wild type AR many ARΔLBD are unable to activate the full panel of androgen-dependent genes [17–19]. As seen in Figure 2 sorafenib was able to inhibit transactivation of the constitutively active, *C*-terminally truncated AR^Q640X^ in a dose-dependent manner. Inhibition was significant at a concentration of 5 μM (downregulation *versus* untreated controls: 30% ± 7%, *p* = 0.012), reaching its maximum at 10 μM (downregulation *versus* untreated controls: 46% ± 8%, *p* = 0.003). The data suggest that sorafenib affects wild type AR and ARΔLBD signalling in a similar way.

### 2.3. Sorafenib Induces Proteasomal Degradation of AR and AR-V Splice Variants in 22Rv1 Cells

There is experimental evidence that kinase inhibitors directed against p42/p44 MAPK, GSK-3β or CDK1 are able to trigger AR-signalling by modulating AR-protein levels [20–22]. Recently, the multikinase inhibitor sorafenib was shown to diminish PSA-levels in LNCaP and its bicalutamide resistant subline LNCaP-Bic. The reduction in PSA-levels was paralleled by a decrease of full length AR [15]. The observation that sorafenib is able to downregulate intracellular AR-levels prompted us to analyze its effects on intracellular ARΔLBD levels. Although transient expression of AR^Q640X^ is largely sufficient to perform reportergene assays (Figure 2), the expression levels of the AR^Q640X^ protein transfected into PC-3 cells are too low to perform a western blot analysis. Therefore we tested the effects of sorafenib on ARΔLBD-levels in 22Rv1 cells, known to express large amounts of the AR-splicing variant AR-V7 [23]. Although AR^Q640X^ and AR-V7 are generated by different mechanisms, both ARΔLBD receptor forms share several common features like identical transactivation and DNA-binding domains, receptor size (AR-V7: 642 amino acids; AR^Q640X^: 640 amino acids), predominant nuclear localization in the absence of androgens and constitutive activity as shown by activation of PSAP1 luciferase reporter plasmid [23].

**Figure 3:**
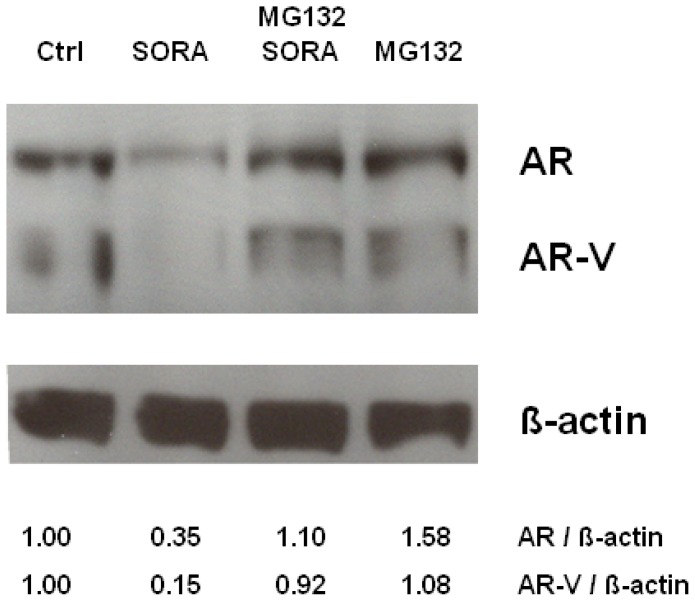
Downmodulation of AR and AR-V in 22Rv1 cells is due to sorafenib induced proteasomal degradation. 22Rv1 cells were incubated with the proteasome inhibitor MG132 (5 μM) for 60 min followed by treatment with sorafenib (5 μM) for 18 h. Subsequently cell extracts were analyzed by Western blot analysis (AR: androgen receptor; AR-V, ARΔLBD generated by alternative splicing; β-actin: loading control; ctrl: untreated control; SORA: sorafenib; MG132: proteasome inhibitor). AR, AR-V and β-actin levels were quantified by densitometry and expressed as fold-change of AR/β-actin or AR-V/β-actin control (ctrl) levels which were set at 1.00.

Interestingly, sorafenib was able to diminish both full length AR as well as AR-V in 22Rv1 cells. Downregulation of AR and AR-V protein levels following sorafenib treatment could be rescued by the proteasome inhibitor MG132, the latter suggesting that sorafenib induces a proteasomal degradation of AR- and ARΔLBD molecules in PCa cells (Figure 3).

### 2.4. Sorafenib Does not Modulate the Subcellular Distribution of AR and ARΔLBD in PCa Cells

**Figure 4:**
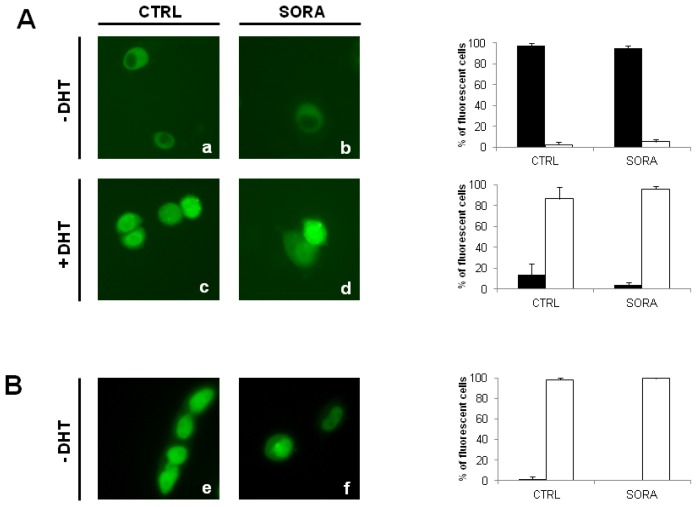
Sorafenib does not modulate subcellular distribution of AR and ARΔLBD in PCa cells. PC-3 cells were transfected with either pAR-t1EosFP or pEGFP-ARQ640X coding for green fluorescent AR-EosFP and ARQ640X-EGFP fusion proteins [17,21]. Cells were treated with sorafenib 5 μM (SORA) in the absence/presence of DHT (10 nM). Intracellular localization of AR-EosFP and ARΔLBD-EGFP was determined by fluorescence microscopy. (**A**) Effect of sorafenib on the subcellular distribution of the AR. Left panel: Fluorescence microscopy of AR-EosFP transfected cells. *Right panel*: Percentage of cells expressing cytoplasmic (black bars) or nuclear fluorescence (white bars), (a) untreated controls; (b) sorafenib; (c) Dihydrotestosterone (DHT); (d) sorafenib + DHT; (**B**) Effect of sorafenib on the subcellular distribution of the ARLBD. Left panel: Fluorescence microscopy of ARΔLBD-EGFP transfected cells. Right panel: Percentage of cells expressing cytoplasmic (black bars) or nuclear fluorescence (white bars); (e) untreated controls; (f) sorafenib.

Besides its effects on AR-stability, various kinase inhibitors were shown to modulate the intracellular localization of the AR [21,24–26]. In consequence we wondered whether the multikinase inhibitor sorafenib is also able to modulate subcellular distribution of AR-molecules. Therefore, we transfected PC-3 cells with expression plasmids coding for green fluorescent AR- and AR^Q640X^-fusion proteins. As seen in Figure 4, sorafenib was unable to influence the subcellular distribution of AR as well as its c-terminally truncated ARΔLBD-counterpart AR^Q640X^.

### 2.5. Inhibition of Cell Proliferation after Sorafenib-Treatment in PCa Cell Lines

**Figure 5:**
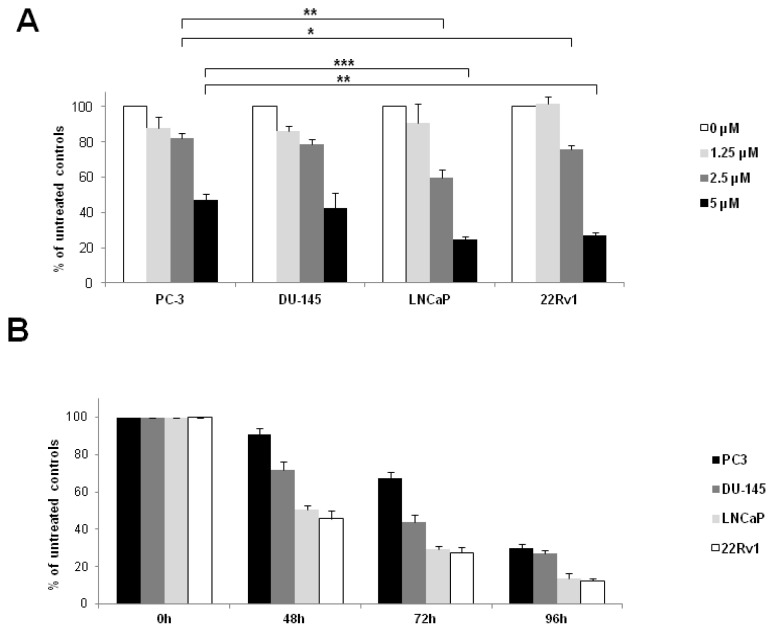
Effect of Sorafenib on prostate cancer cell proliferation. PCa cell lines PC-3 (AR−), DU-145 (AR−), LNCaP (AR+) and 22Rv1 (AR+/AR-V+) were seeded in 96-well plates and allowed to adhere overnight. Subsequently, medium was changed, and cells were grown in RPMI-1640, supplemented with 5% fetal bovine serum and antibiotics in the presence/absence of sorafenib. Cell proliferation was assessed by means of a colorimetric MTT-Assay. (**A**) Dose response curve of sorafenib-induced growth inhibition. PCa cells treated for 72 h with increasing concentrations of sorafenib (0–5 μM). Growth inhibition is expressed as percent of untreated controls, which were set at 100% (* *p* < 0.05; ** *p* < 0.01, *** *p* < 0.001); (**B**) Time course of sorafenib-induced antiproliferative effects in PCa cells. PCa cells treated with sorafenib (5 μM) for different periods of time. Results are expressed as percent of untreated controls, which were set at 100%.

Based on the observation that sorafenib is able to inhibit AR as well as ARΔLBD-signalling we further investigated the antiproliferative effects of the compound on the androgen sensitive LNCaP (AR+) cells, the castration resistant 22Rv1 (AR+, AR-V+) cells as well as the androgen insensitive PC-3 (AR−) and DU-145 (AR−) cells using a MTT cell viability assay [27]. As depicted in Figure 5, the antiproliferative effects of sorafenib were more pronounced in AR-positive or AR/AR-V-positive prostate cancer cells as compared to those lacking the androgen receptor. Differences between AR+ and AR- cells were statistically significant at a sorafenib concentration of 2.5 μM (proliferation rate LNCaP: 60% ± 5% and 22Rv1: 76% ± 3% *versus* PC-3: 82% ± 3%, *p* = 0.002 and *p* = 0.036, respectively).

## 3. Experimental Section

### 3.1. Plasmids and Chemicals

pSG5-AR encoding a wild-type full-length AR (919 amino acids) was supplied by Dr. H. Klocker (Innsbruck, Austria). pAR-t1EosFP coding for a green fluorescent Eos-AR-fusion protein was a generous gift from Dr. F. Oswald (Ulm, Germany). pCruz-ARQ640X and pEGFP-ARQ640X coding for the *C*-terminally truncated ARQ640X (aa 1–640) were provided by and Dr. J. Céraline (Strasbourg, France). The PSA reporter plasmid pPSA-61luc under control of a 6kb-fragment of the human PSA-promoter was a generous gift of Dr. J. Trapmann (Rotterdam, The Netherlands). The artificial ARE(2x) reporter plasmid pLC0548 (pARE(2x)-luc) under control of a synthetic ARE-promoter was created by H. Lebedur and provided by Dr. A. Allera (Bonn, Germany). Renilla reniformis luciferase reporter plasmid (pRL-TK) was purchased from Promega (Mannheim, Germany). Dihydrotestosterone (DHT) and the proteasome inhibitor MG132 were purchased from Sigma-Aldrich GmbH (Taufkirchen, Germany). Sorafenib was a product of LKT Laboratories Inc. (St. Paul, MN, USA). All other chemicals, if not specified, were products of Sigma-Aldrich GmbH (Taufkirchen, Germany).

### 3.2. Cell Culture

PC-3, DU-145, 22Rv1 and LNCaP cells were purchased from the American Type Culture Collection (Manassas, VA, USA). Cell culture was performed as recently described [21].

### 3.3. Reporter Gene Assays

PC-3 cells were transiently cotransfected in 24-well plates with AR-expression plasmids (pSG5-AR; pCruz-Q640X) and different reporter gene constructs (pPSA-61luc, pARE(2x)-luc) using the transfection reagent Polyfect (Qiagen, Hilden, Germany). pRL-TK was cotransfected as an internal control for transfection efficiency. Subsequently, cells were treated with/without 1 nM DHT. After 24 h, reporter gene activity was measured using the Dual-Luciferase Reporter Assay (Promega GmbH, Mannheim, Germany). In this experimental set-up, the PSA- and ARE-reporters are correlated with the effects of the specific experimental conditions, while the activity of the co-transfected constitutive pRL-TK reporter provides an internal control that serves as the baseline response. Normalizing the activity of the experimental PSA- and ARE-reporters to the activity of the internal control minimizes experimental variability caused by differences in cell viability, transfection efficiency, general effects on transcription, translation or protein stability. All experiments were performed as recently described [17].

### 3.4. Nuclear Translocation Assay

PC-3-cells were seeded in 24-well plates and grown in the absence of DHT for 24 h. Subsequently, cells were transfected with pAR-t1EosFP and pEGFP-ARQ640X. After 24 h, cells were treated with ethanol (solvent control), 10 nM DHT, ethanol + 5 μM Sorafenib or 10 nM DHT + 5 μM Sorafenib for 2 h. The fluorescent cells were subsequently counted using a fluorescent microscope as recently described [17,28].

### 3.5. Proliferation Assay

Cell viability was determined by means of a colorimetric MTT assay. This assay is based on the the reduction of tetrazolium salts to formazan derivatives by functional mitochondria. The assay was performed as described by Mosmann [27].

### 3.6. Western Blot Analysis and Immunodetection of AR and ARΔLBD

Total proteins were extracted from cells using RIPA buffer. 40 μg of lysate were separated by Sodium dodecyl sulfate polyacrylamide gel electrophoresis (SDS-Page). Subsequently proteins were transferred onto a PVDF membrane (Invitrolon™ PVDF, Invitrogen, Carlsbad, CA, USA) by semi-dry blotting. AR and ARΔLBD were detected using the monoclonal antibody AR441 (Dako Deutschland GmbH, Hamburg, Germany) at a dilution of 1:2.000 in Tris buffered saline, 0.1% Tween20 (*v*/*v*) (TBS-T). Beta-actin served as a loading control using a mouse monoclonal antibody directed against beta-actin (ab8224, Abcam, Cambridge, UK). Immunoreactive bands were detected using a 1:20.000 dilution of peroxidase-coupled secondary goat anti-mouse IgG (sc-2031, SantaCruz Biotechnology Inc., Santa Cruz, CA, USA) in TBS-T. Signals were visualized by the ECL Plus Chemiluminescent Substrate from Pierce (Rockford, IL, USA).

### 3.7. Statistical Analysis

Data are reported as means ± standard deviations. Statistical significance was determined by an analysis of variance (ANOVA). One-way ANOVA results were confirmed with Bonferroni’s multiple comparison tests. All analyses were performed with the use of SPSS Statistics (version 19.0; SPSS Software, Chicago, IL, USA, 2010).

## 4. Conclusions

Only recently, the efficacy of various tyrosine kinase inhibitors has been evaluated in the treatment of advanced PCa [29–33]. So far, several clinical phase II trials analyzing the impact of a sorafenib-monotherapy on CRPCa reported only moderate therapeutic effects [34–37]. Unfortunately, interpretation of the clinical data generated from these studies is hampered by the relatively low number of patients enrolled in these trials and a limited knowledge about the molecular changes in CRPCa cells following sorafenib treatment.

*In vitro* studies analyzing the precise molecular effects of the multikinase inhibitor sorafenib on CRPCa cells are sparse [38,39]. Recently, Oh *et al*. showed that sorafenib affects AR-signalling in PCa cell lines grown in presence of the synthetic androgen R1881. Inhibition of the canonical AR-signalling pathway after sorafenib treatment was due to a downregulation of AR-levels by an unknown mechanism [15]. To our knowledge, the present study is the first to demonstrate that sorafenib is able to inhibit signalling of *C*-terminally truncated, constitutively active AR variants (ARΔLBD), the latter are thought to be key players in the development of CRPCa. Interestingly, inhibition of AR- as well as ARΔLBD-signalling was paralleled by a sorafenib-induced proteasomal degradation of both receptor types. Consistently, the antiproliferative effects of sorafenib in our experimental model were the most pronounced in AR- or AR/ARΔLBD-positive PCa cells as compared to their AR-negative counterparts (proliferation rate: 22Rv1/LNCaP < PC-3/DU-145). Our finding of a dose-dependent decrease in PCa cell growth after sorafenib treatment is in accordance with previous findings by Oh *et al*. [15]. However, in our study the sorafenib concentrations necessary to induce significant antiproliferative effects were higher than those described by Oh *et al*. At least partially, this may be due to the varying experimental settings used in both studies. Oh and colleagues determined the antiproliferative effect of sorafenib treatment by measuring ^3^H-thymidine incorporation after 48 h in PCa cells that were grown and treated in serum free HITES medium (containing hydrocortisone, insulin, transferrin, estrogen, and selenium). By contrast, in our experiments PCa cells were grown in RPMI-1640 under standard conditions (10% fetal bovine serum, antibiotics), cell viability following sorafenib treatment was determined after 72 h using an MTT-assay which reflects mitochondrial activity by measuring the reduction of tetrazolium salts to formazan derivatives. Therefore it is conceivable that the concentration dependencies may differ in both studies.

In line with our observations, Beardsley *et al*. presented encouraging results using a combination therapy of sorafenib with the nonsteroidal anti-androgen bicalutamide in 39 chemotherapy naïve CRPCa patients. PSA declines or stable disease (≥6 months) were observed in 47% of patients including 26% of patients previously progressing on bicalutamide monotherapy [40].

In summary, the present results suggest that a subset of advanced CRPCa patients, especially those who are expressing AR and/or ARΔLBD, might benefit from a sorafenib treatment. Moreover, strategies to combine multi-targeted kinase inhibitors like sorafenib with hormonal therapies warrant further experimental studies in CRPCa.
